# Effectiveness of COVID-19 Vaccines: Evidence from the First-Year Rollout of Vaccination Programs

**DOI:** 10.3390/vaccines10030409

**Published:** 2022-03-09

**Authors:** Nuno Antonio, Paulo Rita, Pedro Saraiva

**Affiliations:** 1 NOVA Information Management School (NOVA IMS), Universidade Nova de Lisboa, Campus de Campolide, 1070-312 Lisbon, Portugal; prita@novaims.unl.pt (P.R.); pas@novaims.unl.pt (P.S.); 2 Department of Chemical Engineering, CIEPQPF, Universidade de Coimbra, 3030-790 Coimbra, Portugal

**Keywords:** COVID-19, vaccination impacts, data science, statistical models, saving lives

## Abstract

The COVID-19 pandemic has raised a number of new realities, sets of data, and opportunities for data-driven approaches, decisions, and conclusions. One particular area for which developments and data have been made available in record time is related to vaccines and their impacts on health conditions and saving lives. In this article, we use public domain information to study the prevalence of vaccines in different countries and how they can save lives. We conclude that there are different clusters of countries, for some of which solid statistical models were built, and show that vaccination rates provide significant contributions to saving lives in such countries, with impacts that can be computed by simulations based upon these models.

## 1. Introduction

Due to its high level of contagious and rapid geographical spread, COVID-19 was declared a pandemic on 11 March 2020, less than three months after the first cases were diagnosed [1,2].

By the end of November 2020, there were more than 62 million confirmed cases and 1.4 million deaths worldwide [3]. Until then, the pandemic’s impact was mainly correlated with several factors such as the average age of the population, sanitary conditions, health conditions, and public health response restrictions [2]. However, since December 2020, with the rollout of the first vaccination programs [4], the vaccination coverage of the population has become another essential factor in understanding the pandemic evolution, slowing down the progress of the disease, reducing the impact of the pandemic, and saving lives [5].

As recognized by [5] in January 2021, “Vaccines do not save lives; vaccination does”. Despite the efforts of researchers, manufacturers, and governments to produce the first vaccines in a record period of less than one year [4,5], having the vaccines would not suffice by itself. In fact, issues such as dosage, schedules, effectiveness, surveillance, public health response restrictions, and vaccine hesitancy need to be addressed to control the pandemic [5] and save lives effectively.

Vaccine hesitancy, the reluctance and, more often, refusal to have oneself or one’s children vaccinated, is indeed, together with political and logistic issues, one of the main challenges that are influencing the progress of vaccination programs in several countries [6,7,8]. This hesitancy seems to be due to multiple reasons such as age, insurance, confidence in government information, attitude toward vaccines, perceived benefits, and side effects of the vaccine, among others [6,7]. Since vaccination effectiveness requires a large majority of the population to be vaccinated, it depends on each person’s willingness to be vaccinated [5,6,7]. Therefore, it is not enough to overcome vaccine production and distribution’s political and logistical problems. It also becomes imperative to convince everyone to participate in vaccination programs, as otherwise, we will not be able to control the pandemic.

Following previous research on the worldwide impact of COVID-19 [2], this study aims to explore the worldwide influence of vaccination as a tool to control the pandemic and save lives. By providing evidence that vaccines are efficient on a factual data-driven basis, we also hope to contribute to reducing vaccine hesitancy.

## 2. Materials and Methods

Due to its completeness and accessibility to conduct this study, two public datasets made available by Our World in Data (OWID) [9] were used. One dataset with global data and one specific to vaccination by the manufacturer. OWID is a project from the Global Change Data Lab, a non-profit organization based in the United Kingdom. The dataset was collected on 1 December 2021. It includes worldwide daily COVID-19 related data, covering confirmed cases and deaths, hospital infirmaries and Intensive Care Units (ICU), policy responses, tests and vaccination rates, demographics, among other types of data. The complete list of variables available is detailed in Table A1 and Table A2.

The analysis and modeling of the data were performed in Python, following the Cross-Industry Process Modeling for Data Mining (CRISP-DM) framework [10], using standard packages employed in Data Science like NumPy [11], Pandas [12], Matplotlib [13], and Seaborn [14].

### 2.1. Data Understanding

Summary statistics (Table 1) show that the used global dataset contains 127,297 observations, spanning from 1 January 2020 to 30 November 2021, and hence does not yet reflect any of the new COVID-19 dynamics related to the new Omicron variant.

**Figure 1:**
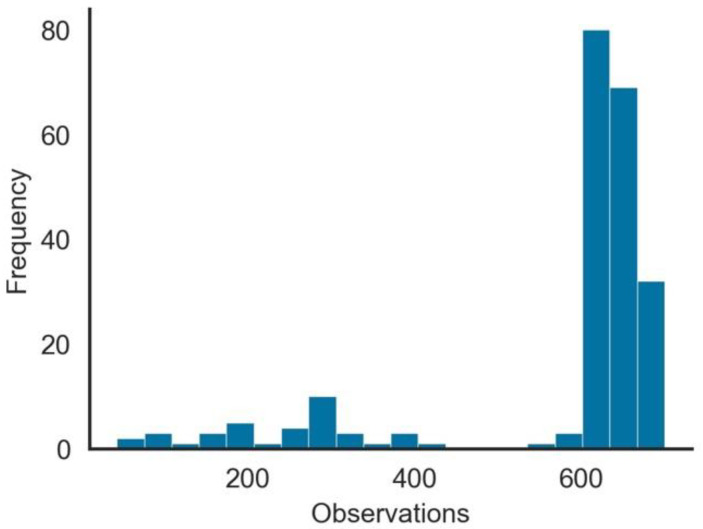
Histogram of numbers of days with vaccine observations per GPE.

**Figure 2:**
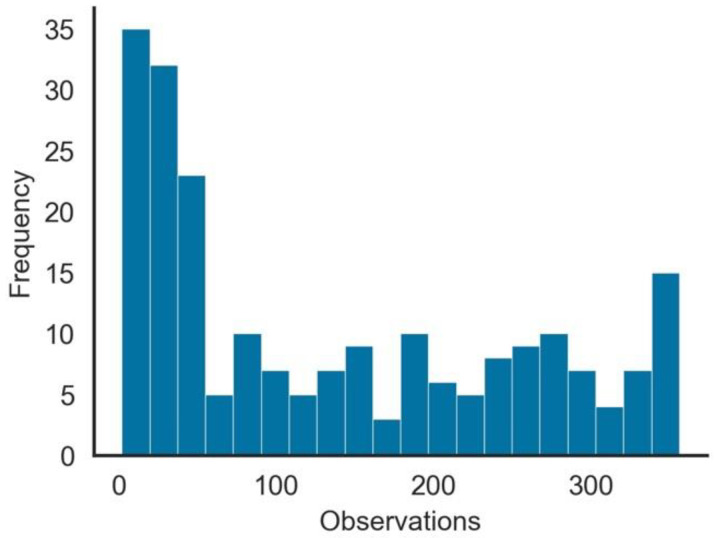
Histogram of vaccination cumulative numbers of days per GPE.

Summary statistics also show that there are some data quality issues related to this dataset, such as the following:

- Except for *iso_code*, *continent*, *location*, and *date*, all other variables had missing values. The range of missing values varied from 5.7% in *total_cases* to 96.3% in *excess_mortality*. Variables that could be important in measuring the vaccination impact, such as *weekly_icu_admissions* and others, had a high proportion of missing values.
- The number of observations per Geo-Political Entity (GPE), as seen in Figure 1, was highly skewed. From the 222 GPEs in the dataset, over 100 had more than 500 days of observations, while for other GPEs, the number was substantially low.
- As expected, since vaccination did not start simultaneously in all GPEs, the number of observations per GPE with *people_vaccinated* > 0 was not uniform. As shown in Figure 2, although many GPEs had more than 100 days of vaccination, in more than 50 GPEs, the number of days since it started was less than 50 days. From the 222 GPEs, five did not even present any vaccination numbers. Contrastingly, 25% of the GPEs had vaccination data for more than 243 days.
- Some GPEs did not consistently provide reports, i.e., reporting later after the beginning of the pandemic or even not reporting during some days.
- Gibraltar presented values of *people_vaccinated_per_hundred* above 100% (121.43%), which may indicate that the *population* value was incorrect or that there were vaccinated persons that were not part of its population.

Only a few GPEs systematically report their vaccination by the manufacturer. For that reason, the vaccination by manufacturer dataset only includes data from 39 GPEs. As presented in Table A2, this dataset has the total of vaccines administered by day or week (GPEs report in daily or weekly intervals).

### 2.2. Data Preparation

Due to the high number of columns (variables) with missing values, it was decided to use only GPEs without missing values in the columns *continent*, *location*, *date*, *total_deaths_per_million*, *total_cases_per_million*, *people_vaccinated_per_hundred*, *people_fully_vaccinated_per_hundred*, *population_density*, *median_age*, *aged_70_older*, *gdp_per_capita*, *cardiovasc_death_rate*, *diabetes_prevalence*, *life_expectancy*, *human_development_index,* and *stringency_index*, after 1 March 2020 (March 2020 was the month when the pandemic was declared). This variable selection resulted in the removal of data from mostly small or less developed GPEs: Andorra, Anguilla, Antigua and Barbuda, Armenia, Aruba, Bermuda, Bonaire Sint Eustatius and Saba, British Virgin Islands, Cayman Islands, Comoros, Cook Islands, Cuba, Curacao, Dominica, Equatorial Guinea, Eritrea, Faeroe Islands, Falkland Islands, French Polynesia, Gibraltar, Greenland, Grenada, Guernsey, Guinea-Bissau, Hong Kong, Isle of Man, Jersey, Kiribati, Kosovo, Liechtenstein, Macao, Maldives, Marshall Islands, Micronesia, Monaco, Montenegro, Montserrat, Nauru, New Caledonia, Niue, North Macedonia, Northern Cyprus, Palau, Pitcairn, Saint Helena, Saint Kitts and Nevis, Saint Lucia, Saint Vincent and the Grenadines, Samoa, San Marino, Sao Tome and Principe, Serbia, Sint Maarten (Dutch part), Solomon Islands, Somalia, South Sudan, Syria, Taiwan, Tokelau, Tonga, Turkmenistan, Turks and Caicos Islands, Tuvalu, Vatican, Wallis, and Futuna.

To compare values between before and after vaccination, two additional modeling datasets were created: Before–data from 30 November 2020; After–data from 30 November 2021 However, since all the above-mentioned numeric variables represented accumulated values or values at the current moment, the column *stringency_index* was replaced in these datasets with the median of the *stringency_index*. This way, instead of having a variable with the level of public health measures from 30 November 2020 and 30 November 2021, we used a variable with the median from the beginning of the pandemic until the last date of the collected dataset (*stringency_index_med*). Nevertheless, since there were GPEs with missing values in the variable *stringency_index*, the observations of those GPEs were also removed. The removal of those observations resulted in reducing these two modeling datasets (Before and After vaccination) to information for about 159 GPEs.

As for the vaccination by the manufacturer, data was only available for the last week of November for 35 GPEs. Therefore, data for the other 2 GPEs was removed. Considering that not all GPEs reported daily data, the “after” vaccination data was selected from 26 November 2021. This date corresponds to when all GPEs had data available in our information sources.

### 2.3. Clustering Model

**Figure 3:**
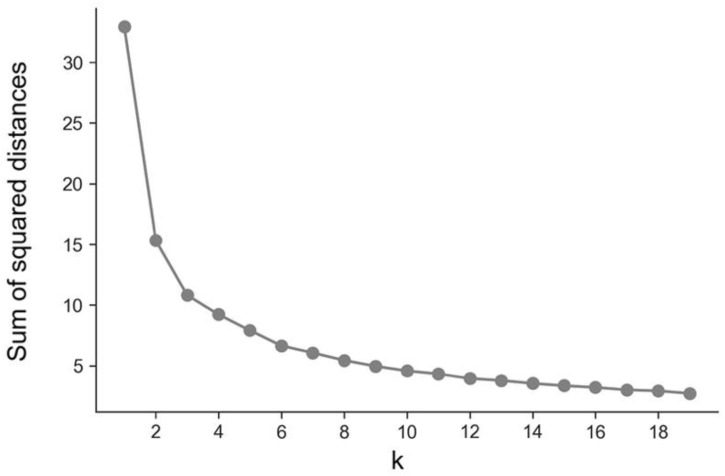
K-means k-selection plot (Elbow method) for clusters of GPEs.

To study how the 159 GPEs mentioned above were grouped on 30 November 2021, a clustering model was built using the k-means algorithm. The “elbow” method was employed to select the number of clusters (k). As presented in Figure 3, the decrease of the sum of the squared distances slowed at k = 3. Therefore, it was decided to consider the existence of three clusters of GPEs (named A, B, and C).

### 2.4. Regression Model

To study the factors that could explain the number of deaths and how vaccination impacted the severity of cases, we opted to build a regression model that can be easily interpreted. This model was built using the ordinary least squares regression algorithm implemented in the statsmodels package [15]. The dependent variable in the model was a new variable, named *death_ratio*, a simple ratio of the total number of deaths over the total number of cases.

**Figure 4:**
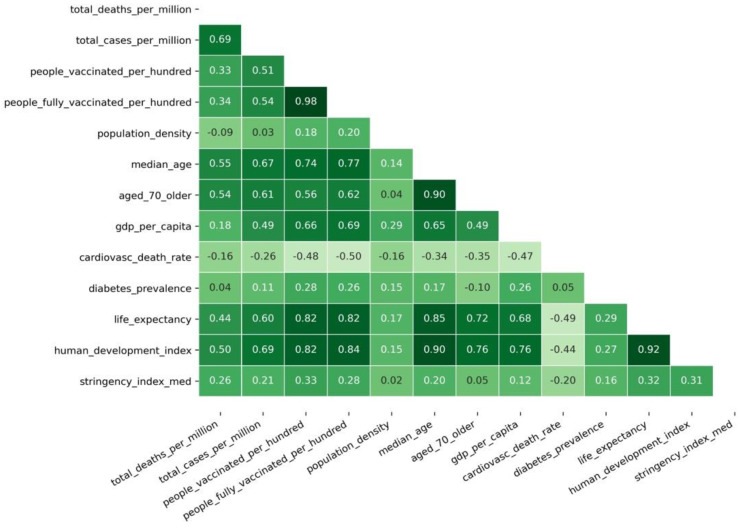
Variables’ Pearson correlation matrix.

The correlation between features in the “After vaccination” modeling dataset was first analyzed to build this model. As depicted in Figure 4, the analysis showed that several variables, as expected, have high correlation values. Therefore, it can be considered redundant for regression purposes, such as *people_vaccinated_per_hundred* and *people_fully_vaccinated_per_hundred* with a 0.98 correlation coefficient. Other variables were tested as independent variables, namely the human development index (HDI) (*human_development_index*). The HDI is built from multiple indicators such as life expectancy, gross income per capita, among others. This composition explains the high correlations between *human_development_index* and *life_expectancy*, *median_age*, *aged_70_older*, and *gdp_per_capita*. However, since there was a high correlation between HDI and the vaccination percentage of the population, it was decided to use only the variable *death_ratio* as the dependent variable and *people_vaccinated_per_hundred* (PV) as the independent variable in the regression model. This regression dataset was a construct derived from the original dataset, where the unit of analysis corresponds to the weeks from December 2020 to November 2021. To create the dataset, the average value of the two variables was computed per cluster and week. Because the data relationships were found to be not linear, we also added one variable that was a degree polynomial of the PV variable. Adding additional polynomials was revealed unnecessary as they did not improve the models’ statistical quality.

## 3. Results and Discussion

This section provides the main results obtained from our data analysis, incorporating the discussion of the corresponding most important findings.

### 3.1. Descriptive Analysis

Figure 4 shows that besides the expected high positive correlation between *people_vaccinated_per_hundred* and *people_fully_vaccinated_per_hundred*, *aged_70_older* and *median_age*, *life_expectancy*, and *median_age*, there were other interesting patterns in terms of bivariate correlations. In addition to the high positive correlations between *human_development*_index and variables used to create that index (*gdp_per_capita*, *life_exprectancy*, and others), there was a highly positive correlation between *human_development_index* and *total_cases_per_million* (0.69). Given that more developed GPEs had a higher median age and higher income [16], this positive correlation values suggest that more developed GPEs tend to report more cases than other GPEs. However, the correlations between *human_development_index* and *total_deaths_per_million* (0.50) and between *human_development_index* and *people_vaccinated_per_hundred* (0.82) indicate that although developed GPEs tend to report more cases, they also have proportionally fewer deaths, possibly due to their higher vaccination rates. The positive correlation between *total_cases_per_million* and both *people_vaccinated_per_hundred* and *people_fully_vaccinated_per_hundred* (around 0.50) seems to indicate that more cases are associated with higher vaccination rates. However, this high correlation reinforces the idea that GPEs with less vaccinated people are also less likely to report cases correctly and accurately, with smaller COVID-19 tests being conducted.

**Figure 5:**
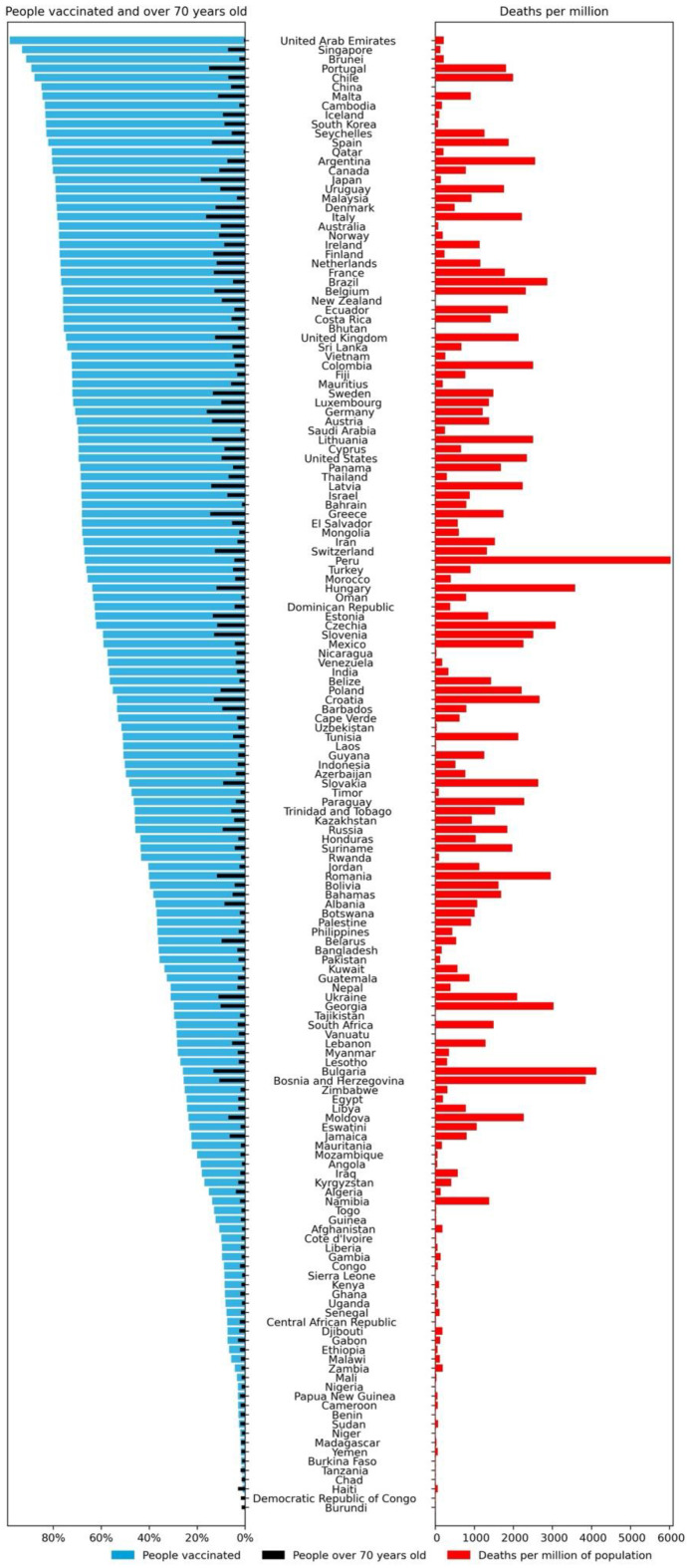
Percentage of people vaccinated and percentage of people over 70 years old versus deaths per million of the population.

The relatively high correlation between *people_vaccinated_per_hundred* and *people_fully_vaccinated_per_hundred* with *median_age*, *aged_70_older*, *gdp_per_capita*, and *human_development_index*, with values between 0.56 and 0.84, also points to a direct relationship between the vaccination rate and the development level of the GPE. A visualization of the percentage of the vaccinated population and percentage of people over 70 years old versus the total of deaths per million of the population can be seen in Figure 5. As shown, there is a clear contrast between the top, middle, and bottom of this visual representation. At the top, we can find primarily developed GPEs, as can be asserted by the percentage of the population over 70 years old in those GPEs. These GPEs, at the top of Figure 5, present the higher vaccination rates but, in general, not so many deaths as the GPEs in the middle of the figure, which have lower vaccination rates than the ones at the top and tend to present more deaths. The vaccination efficacy may explain this tendency. The bottom of the figure is composed mainly of less developed GPEs with very low vaccination rates. Conversely, these were also GPEs with smaller numbers of deaths. This fact may be yet one more indication that these GPEs are not enforcing an adequate COVID-19 monitorization and reporting policy.

**Figure 6:**
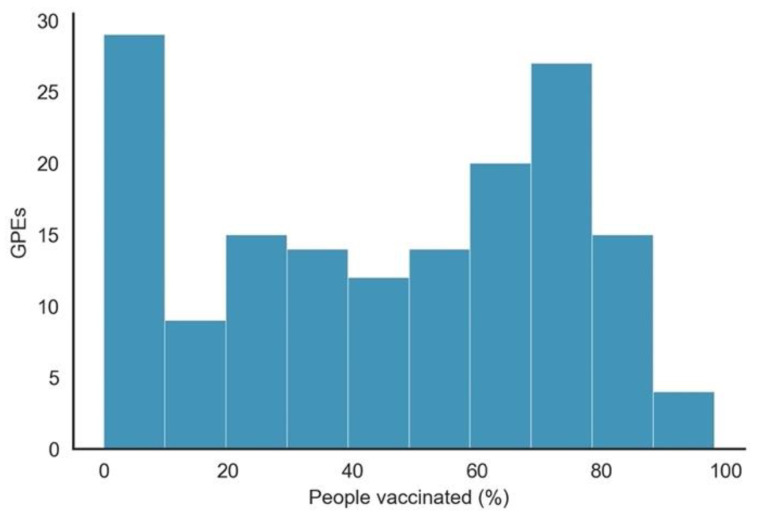
Histogram of the percentage of the population vaccinated by GPEs.

The abyss between the percentage of the population vaccinated between GPEs can be confirmed in Figure 6. While over 30 GPEs had vaccinated less than 20% of their population, over 35 GPEs had vaccinated more than 70%.

**Figure 7:**
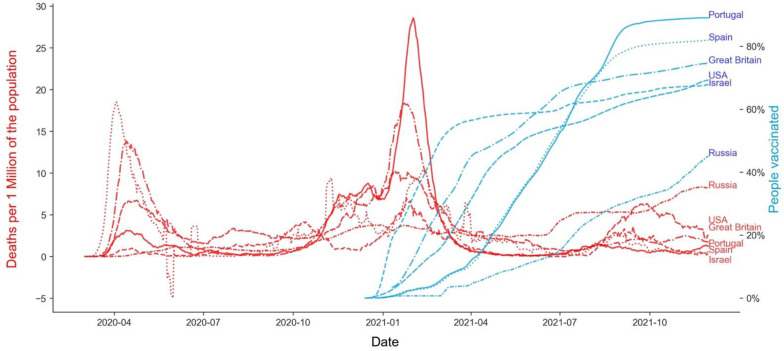
Seven-day moving average of daily deaths per million of the population vs. seven-day moving average percentage of the population vaccinated (examples from GPEs with similar development level vaccination start dates and profiles).

Another demonstration of the vaccination effect can be seen in Figure 7, which illustrates the daily evolution of the pandemic by plotting the seven-day moving average of daily deaths versus the seven-day moving average of the percentage of the vaccinated population. Since plotting this information for all the GPEs under study would not produce an interpretable visualization, we decided to show here only six particular GPEs: Israel, Great Britain, Portugal, Russia, Spain, and the USA. These GPEs were chosen due to their development level and the start of vaccination similarity. As distinctly seen, over time, as a higher percentage of the GPEs’ population is vaccinated, the number of deaths tends to decrease or stabilize, particularly when the rate of the vaccination reaches values above 60% of the population.

As illustrated in Figure 7, the relationship between vaccination rates and lives saved is not linear and can also depend on the vaccines being provided to the population. There seems to be a minimum threshold of around 20% for the vaccination rates to be converted into significant death decreases, followed by a rapid decrease of deaths per capita and then a relatively stable situation below five daily deaths per million people.

The resulting clustering model was not balanced in terms of the number of GPEs in each cluster. While cluster A was composed of 37 GPEs, cluster B was composed of 69 and cluster C of 53 GPEs.

**Figure 8:**
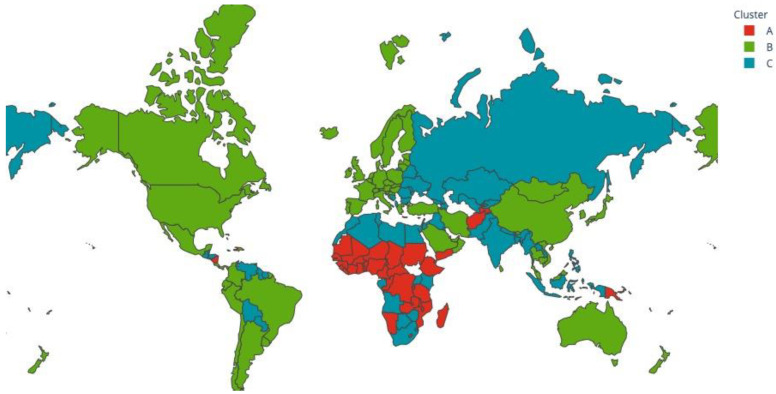
GPEs’ clustering geographic representation.

The analysis of the mean values of the different variables per cluster, as detailed in Table 2, shows that there may be three distinct clusters of GPE. In cluster A, we find the GPEs where COVID-19 had a higher reported health impact, with more deaths per cases (higher *death_ratio*). This cluster comprises mainly less developed GPEs, as seen in the variable *humand_development_index*. These were the GPEs that implemented less restrictive healthcare measures (*stringency_index_med*). This application of less restrictive measures could also be related to stronger economic needs. This lack of economic capacity could also explain the lower vaccination percentage in this cluster (9.6%). As seen in Figure 8, the GPEs of cluster A were primarily from Africa and the Middle East.

In contrast, we have cluster B, where COVID-19 had a smaller impact in terms of deaths by cases. Cluster B is formed by the higher developed GPEs. As shown in Figure 8, Cluster B is composed predominantly of European, North and South American, richer Asian, and Oceanian GPEs. Lastly, in cluster C, we find the “not-so-developed” GPEs. These GPEs had a higher number of deaths per cases. Still, much inferior to the impact found in cluster A. Geographically, as shown in Figure 8, these are primarily GPEs from Latin America, north Africa, and Asia. Reversely to other indicators, the stringency index in cluster C is higher than in clusters A and B, thus suggesting that since GPEs in this cluster did not have the vaccination capability of GPEs of cluster B, they may have opted for higher levels of public health restrictions.

When comparing the probability of dying in the case of contracting the virus before the vaccination programs rollout (30 November 2020), or in other words, the odds of dying from COVID-19 in the case of contracting the virus, as presented in Table 3, it was between 1.8% and 2.8% across the clusters. However, that probability was substantially reduced after vaccination. Before vaccination, people from the GPEs of cluster B, higher developed GPEs, and as such, with an older population less capable of surviving the disease, had a probability of dying of 2.72%. In cluster A that probability was 2.04%, and in cluster B of 1.74%. Notwithstanding, after vaccination, cluster B turned from being the cluster with the highest probability of dying to being the one with the lowest (1.36%). This decrease means that in the one year of vaccination, the probability of dying in case of testing positive decreased 0.32 percentual points (pp) in cluster A, 1.36 pp in cluster B, and 0.18 pp in cluster C. The odds ratio shows that in cluster B, the cluster with higher vaccination rates, there was a 50.7% decrease in the odds of dying compared to the same day in the previous year. However, in cluster C, the cluster with the second-highest vaccination rate, the decrease was only 11.1%. In cluster A, the cluster of GPEs with the lowest vaccination rates, the decrease of the odds of dying was only 16.3%. These results emphasize the impact of vaccination in reducing the number of deaths.

**Figure 9:**
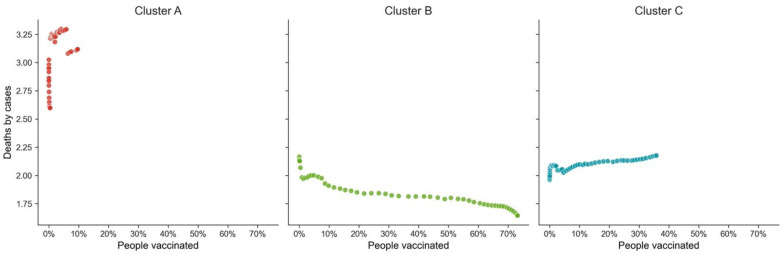
Weekly deaths by cases vs. percentage of the population vaccinated. Cluster A is the cluster with the highest impact. Cluster B, cluster with smaller impact in terms of deaths by cases. Cluster C, cluster with second-highest vaccination rate and second-highest death rate.

The difference between clusters is even more evident when analyzing the average deaths by cases (*death_ratio*) by the average vaccinated percentage of the population per week (Figure 9). While in cluster B, it is possible to see a pattern where the increase in vaccination resulted in a decrease in the *death_ratio*; the opposite happened in cluster C. As vaccination increased, *death_ratio* also increased. In cluster A, there seemed to also be some sort of discontinuity in the *death_ratio* time profile evolution. These observations seem to show once more that only when values above 20% of the population vaccinated were reached did there emerge a stable pattern of saving lives, leading to values below 1.75 deaths per 100 cases of COVID-19.

**Figure 10:**
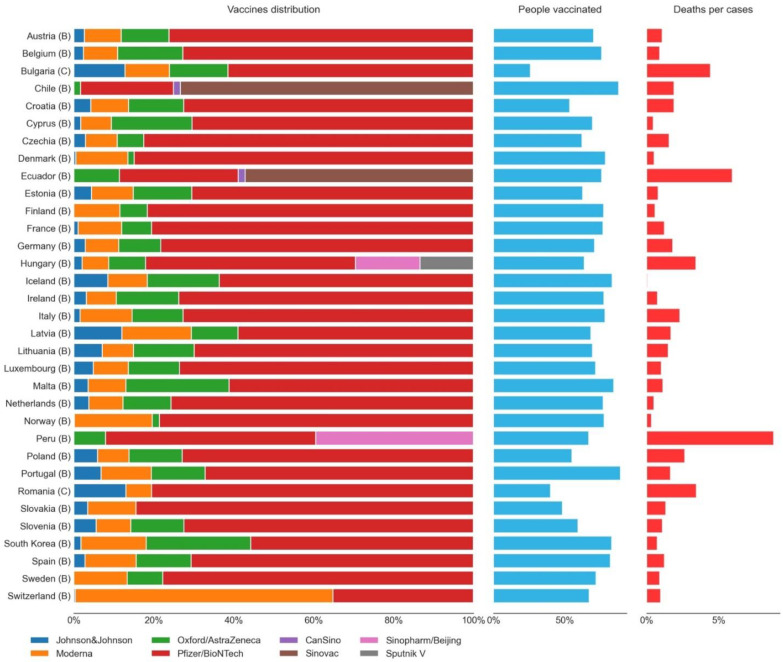
Vaccines by manufacturer distribution and percentage of people vaccinated on 26 November 2021, and percentage of deaths by cases since 12 January 2020, by GPE. The cluster of each GPE is presented in parenthesis.

One possible explanation for the different trends in the two clusters with a higher percentage of vaccinated people (clusters B and C) could be the types of vaccines that were mainly administrated in each country. However, as shown in Figure 10, due to the limitations and types of available data, inference on the efficiency of the different types of vaccines is hard to make. This limitation makes this particular topic something that may be studied in more detail as part of future work and further analysis. For instance, the data now available only includes the number of doses administrated. Since some vaccines were of a single dose, it is expected for this representation of such vaccines over others to be underrated. Secondly, most GPEs that provided data by vaccine manufacturers were from cluster B, many of which were from the European Union; therefore, having followed somewhat more similar vaccination policies.

Notwithstanding these limitations, it is possible to see in Figure 10 that Bulgaria and Romania, two European GPEs from cluster C, are among the GPEs with higher death ratios after the vaccination started, despite their low vaccination rates (compared to the other GPEs). Since the distribution of vaccines by manufacturers in Bulgaria and Romania was not much different from other European Union members, the higher death ratio seemed to be related to the lower vaccination rate of these GPEs. Among the GPEs represented in Figure 10, the ones that show a clear, distinct pattern of vaccination by the manufacturer are Chile, Ecuador, Hungary, and Peru. All of these GPEs are from cluster B. Except for Chile, the remaining three GPEs are among the top five countries with a higher death ratio. A lower vaccination rate could explain this high death ratio. However, that is not the case. Despite having higher death rates, Ecuador, Hungary, and Peru are between these 33 GPEs the 19th, 9th, and 10th in terms of lower vaccination rates, respectively. The development level of these GPEs may also have contributed to the higher death ratio values that were found. Nevertheless, since Ecuador, Hungary, and Peru administered some types of vaccines that the remaining GPEs did not, this raises the question of the possible different effectiveness real-life performances of some of the vaccines, something that may deserve additional research work to be conducted in the future when more public domain data becomes available in this regard.

### 3.2. Regression Model

The global fitted regression model of the ratio of deaths by cases was the following:

(1) $$\begin{array}{c} death\_ratio=2.3347-0.5973\times PV+0.2933\times PV^{2}\\ R^{2}=0.355,\;F(2,156)=42.97,\;p\;<\;0.000 \end{array}$$

This overall regression did not present very interesting statistically significant results. However, regression models built for each cluster produced significantly improved statistical models as expected, and shown below:

(2) $$\begin{array}{c} death\_ratio_{A}=3.0688+0.4676\times PV-0.3933\times PV^{2}\\ R^{2}=0.533,\;F(2,50)=28.58 \end{array}$$

(3) $$\begin{array}{c} death\_ratio_{B}=1.8516-0.2163\times PV+0.0974\times PV^{2}\\ R^{2}=0.889,\;F(2,50)=199.4 \end{array}$$

(4) $$\begin{array}{c} death\_ratio_{C}=2.0838+0.0754\times PV-0.0292\times PV^{2}\\ R^{2}=0.726,\;F(2,50)=66.28 \end{array}$$

As depicted in Figure 9, the dissimilarity among clusters does justify the impossibility of building a good single global regression model to explain deaths per case (1). However, as also suggested by Figure 9, statistically significant models can help understand the power of vaccination in reducing the number of cases by cluster, particularly in clusters B and C.

To further study by simulation from the above models the impact of vaccination on saving lives, we applied the regression models (3) to the respective clusters in the week of 28 November 2021, considering a scenario with the vaccination variable having an increase of 5%. Therefore, simulating that in the week between 21 and 28 November, it would have been possible to increase vaccination rates by 5%. As shown in Table 4, it would then have been possible to save around half a million lives in cluster B GPEs, the ones where significant vaccine rates have already been achieved.

**Figure 11:**
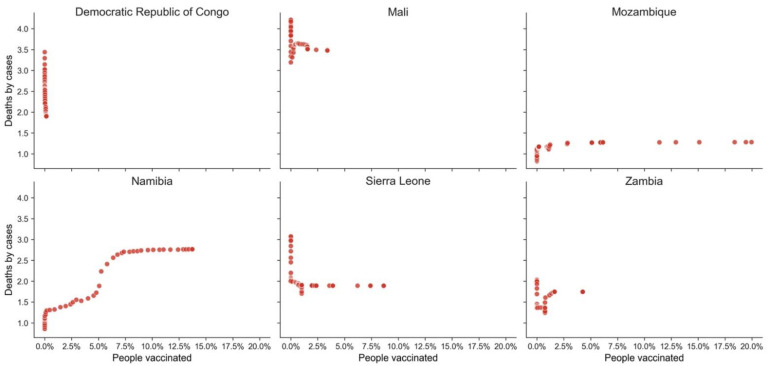
Weekly deaths by cases vs. percentage of the population vaccinated (some GPEs from cluster A).

**Figure 12:**
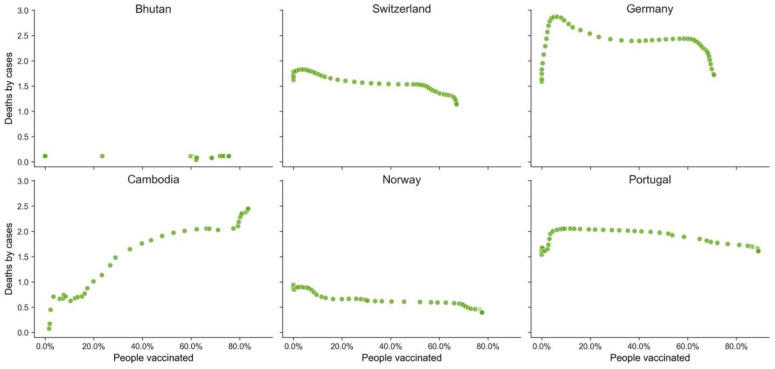
Weekly deaths by cases vs. percentage of the population vaccinated (GPEs from cluster B).

**Figure 13:**
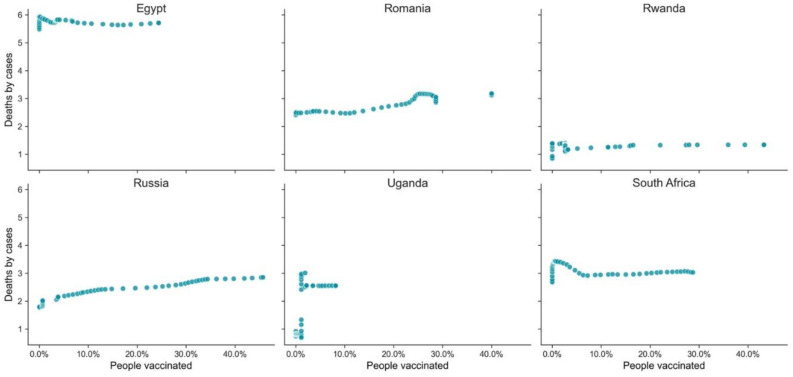
Weekly deaths by cases vs. percentage of the population vaccinated (GPEs from cluster C).

Vaccination does have a significant impact and potential for saving lives, as illustrated above, but it is not the only factor that increases the probability of not dying from COVID-19. When we analyze examples of GPEs from the different clusters (Table 5, Table 6 and Table 7, Figure 11, Figure 12 and Figure 13), distinctive behaviors in the three clusters can be found. There was a high variance in the death ratio in cluster A, independently of HDI and vaccination rate. In cluster A, the weekly profiles of death ratio by vaccination rate were very erratic and GPE specific, as shown in Figure 11. In cluster B, even though the cluster was composed of GPEs with a wide range of HDI, there seems to be a pattern over time of decrease of the death ratio as the vaccination rate increases (see Figure 9 and Figure 12). This pattern, as previously mentioned in Figure 7, seems to be more robust when vaccination rates over 60% are reached. However, there are also exceptions, such as Bhutan and Cambodia, two of the less developed GPEs in cluster B. Cluster C weekly results are indeed the stranger, even when looking at some examples with different HDI and vaccination rates (Figure 12). Most GPEs in cluster C did not show a decrease in the death ratio, despite the increase of the vaccination rates. This situation shows that there seems to be a minimum threshold value of vaccination rates to make visible its statistical impacts on deaths and on saving lives.

## 4. Strengths and Limitations

One of the most significant strengths of this study is the use of one-year public domain data from a large number of GPEs. Since not all GPEs had the same easiness of access to vaccines and not all GPEs are at the same level of development, vaccination did not have the same impact over all of the GPEs considered. However, it was possible to see that in developed GPEs with good vaccination coverage, vaccination was, unequivocally, an effective weapon against COVID-19. Another strength of this study is that it uses publicly available data from OWID, which is continuously updated. That being the case, any researcher can reproduce this study and even extend or expand it as time goes by and more data becomes available. This study also shows how data science fields and methods, namely statistics, machine learning, and data visualization, combined together, can be used to better understand complex phenomena, pandemic behavior, and vaccination impacts.

This study is not without limitations, some of which are summarized next. Since the main objective was to give a global perspective of the vaccination impact, we tried to analyze data from as many GPEs as possible and in as many dimensions as possible. However, one important dimension that was not analyzed yet, given data collection constraints (namely, lack of available data), concerns the stratified impacts and conclusions associated with each specific COVID-19 virus variant. Such stratified impacts, including specifically, the effectiveness of vaccines depending on the pandemic prevalence or deaths by variants (e.g., the Delta VOC), could bring an additional perspective on vaccination impact and effectiveness. Similarly, more detailed analysis can be considered regarding stratified data analysis and modeling according to the kinds of vaccines administered in different countries.

Notwithstanding, to perform such additional studies, more detailed data needs to be collected and made available for many of the studied GPEs. More detailed data would ensure that a similar global worldwide analysis could be conducted, along the perspective adopted in this paper but relying on more detailed data that is not yet available at this stage. To overcome some of these data limitations, we also ended up removing some GPEs from our data analysis and modeling efforts, mostly smaller GPEs with high numbers of missing values.

In fact, this lack of broader stratified quality data and the lack of certain types of detailed data, such as the vaccines administered by manufacturers, is something all GPEs should be committed to addressing in order to allow for more detailed data analysis to be conducted in future studies. Only by making more of this good and detailed quality data available will data science be able to study further and learn from what has happened, understand why it happened, and contribute to better decision-making and future improvements in handling the COVID-19 or other pandemics.

Something that may also influence the outcome of this and future studies and that GPEs should try to establish for handling pandemics regards the adoption of standardized criteria to define what are COVID-19 or other virus-related deaths. For example, some GPEs consider any COVID-19 positive hospitalized patient who died, independently of the reason the patient was hospitalized as a “COVID-19” death. Others did not adopt the same criteria. Therefore, this raises additional issues about data comparison, model building, or an overall consistent data-driven approach to decision-making and understanding of the phenomena across the world or in different GPEs.

## 5. Conclusions

This paper aimed at identifying the effectiveness of the vaccination programs against the COVID-19 virus during their first year of implementation. Vaccination and, even more so, high vaccination rates have played a pivotal role in saving lives. On the one hand, it significantly reduced the need for infirmary as well as intensive care unit hospitalizations. On the other hand, deaths per cases went down substantially. Findings support these conclusions not only when we address data reporting reality before and after vaccination but also across clusters of GPEs.

One should note that cluster B, with the higher developed GPEs, was the only one showing a more robust model since it was the unique cluster with more comprehensive data and evolution regarding the rollout of the vaccination programs. Moreover, despite its more accurate reality check in terms of much more COVID-19 tests, leading to the identification of much more cases, aggravated by its higher exposure to the pandemic consequences due to its aging population, cluster B was the one where vaccination won big over the virus. This evidence envisages the urgent need to ensure clusters A and C follow suit of what has already been achieved in cluster B.

As a follow-up on the results found in this paper, future research might consider a detailed analysis of the different variants, namely the Delta variant and the recent Omicron variant, due to their higher contagious rates and apparent less seriousness in terms of hospitalizations. It will also be interesting to study both clusters A and C when they reach much higher vaccination rates and compare them with cluster B vaccination rates already achieved by these GPEs. Further, a few GPEs, such as Cambodia, have shown quite unexpected results. Despite achieving vaccination rates over 50%, their deaths per case do not fall significantly, hence recommending new research to be performed in the future. Since the currently available data suggest that some vaccines may not be as efficient as others, further research should also study the relationship between the percentage of vaccinated people, the types of vaccines administered, and GPEs’ development level.

## Figures and Tables

**Table 1:** Dataset summary statistics (*iso_code* and *date*).

Variable	Count	Min.	Max
*iso_code*	127,297	-	-
*date*	127,297	1 January 2020	30 November 2021

**Table 2:** Mean values per cluster on 30 November 2021.

	A	B	C
*people_vaccinated_per_hundred*	9.637	73.227	35.795
*human_development_index*	0.516	0.853	0.705
*stringency_index_med*	39.465	58.468	63.640
*death_ratio*	3.117	1.645	2.176

**Table 3:** Probabilities and odds of dying in case COVID-19 is contracted.

		A	B	C
*Before vaccination* *(30 November 2020)*	Probability of dying	0.0204	0.0272	0.0174
	Odds of dying	0.0209	0.0280	0.0178
*After vaccination* *(30 November 2021)*	Probability of dying	0.0172	0.0136	0.0156
	Odds of dying	0.0175	0.0138	0.0158
*Odds ratio of dying (between the two periods)*		0.8373	0.4931	0.8895
*p-value of the odds ratio of dying (between the two periods)*		<0.01	<0.01	<0.01
*Difference in the odds ratio of dying (between the two periods)*		0.16237	0.5069	0.1105

**Table 4:** Estimation exercise for the last week of November 2021 in Cluster B GPEs.

		Cluster B
Week 28 November 2021	Deaths	3,533,115
	Cases	176,273,398
	Death ratio (%)	1.649
	Vacc. (%)	73.092
Estimation Additional 5% increase in vaccination	Death ratio (%)	1.709
	Deaths	3,012,163
	Vacc. (%)	78.092
	Saved people	520,952

**Table 5:** Example of GPEs from cluster A with different HDI, deaths ratio, and vaccination rate on 30 November 2021.

GPE	HDI	Death Ratio	People Vaccinated %
Democratic Republic of Congo	0.480	1.901	0.15
Mali	0.434	3.476	3.40
Mozambique	0.456	1.281	19.97
Namibia	0.646	2.766	13.73
Sierra Leone	0.452	1.890	8.64
Zambia	0.584	1.745	4.26

**Table 6:** Example of GPEs from cluster B with different HDI, deaths ratio, and vaccination rates on 30 November 2021.

GPE	HDI	Death Ratio	People Vaccinated %
Bhutan	0.654	0.114	75.54
Switzerland	0.955	1.136	67.04
Germany	0.947	1.718	70.81
Cambodia	0.594	2.447	83.47
Norway	0.957	0.394	77.56
Portugal	0.864	1.607	89.04

**Table 7:** Example of GPEs from cluster C with different HDI, deaths ratio, and vaccination rate on 30 November 2021.

GPE	HDI	Death Ratio	People Vaccinated %
Egypt	0.707	5.710	24.40
Romania	0.828	3.176	40.03
Rwanda	0.824	1.337	43.27
Russia	0.543	2.850	45.74
Uganda	0.544	2.550	8.15
South Africa	0.709	3.027	28.75

## Data Availability

The data employed in this studio can be found at the OWID GitHub repository at https://github.com/owid/COVID-19-data (accessed on 1 December 2021).

## Appendix A

Table A1 details the content of the OWID dataset.

**Table A1:** OWID global dataset dictionary.

Variable	Type	Description
*total_cases*	Confirmed cases	Total confirmed cases of COVID-19
*new_cases*	Confirmed cases	New confirmed cases of COVID-19
*new_cases_smoothed*	Confirmed cases	New confirmed cases of COVID-19 (7-day smoothed)
*total_cases_per_million*	Confirmed cases	Total confirmed cases of COVID-19 per 1,000,000 people
*new_cases_per_million*	Confirmed cases	New confirmed cases of COVID-19 per 1,000,000 people
*new_cases_smoothed_per_million*	Confirmed cases	New confirmed cases of COVID-19 (7-day smoothed) per 1,000,000 people
*total_deaths*	Confirmed deaths	Total deaths attributed to COVID-19
*new_deaths*	Confirmed deaths	New deaths attributed to COVID-19
*new_deaths_smoothed*	Confirmed deaths	New deaths attributed to COVID-19 (7-day smoothed)
*total_deaths_per_million*	Confirmed deaths	Total deaths attributed to COVID-19 per 1,000,000 people
*new_detaths_per_million*	Confirmed deaths	New deaths attributed to COVID-19 per 1,000,000 people
*new_deaths_smoothed_per_million*	Confirmed deaths	New deaths attributed to COVID-19 (7-day smoothed) per 1,000,000 people
*excess_mortality*	Excess mortality	Percentage difference between the reported number of weekly or monthly deaths in 2020–2021 and the projected number of deaths for the same period based on previous years
*excess_mortality_cumulative*	Excess mortality	Percentage difference between the cumulative number of deaths since 1 January 2020 and the cumulative projected deaths for the same period based on previous years
*excess_mortality_cumulative_absolute*	Excess mortality	Cumulative difference between the reported number of deaths since 1 January 2020 and the projected number of deaths for the same period based on previous years
*excess_mortality_cumulative_per_million*	Excess mortality	Cumulative difference between the reported number of deaths since 1 January 2020 and the projected number of deaths for the same period based on previous years, per million people
*icu_patients*	Hospital and ICU	Number of COVID-19 patients in intensive care units (ICUs)
*icu_patients_per_million*	Hospital and ICU	Number of COVID-19 patients in intensive care units (ICUs) per 1,000,000 people
*hosp_patients*	Hospital and ICU	Number of COVID-19 patients in hospital
*hosp_patients_per_million*	Hospital and ICU	Number of COVID-19 patients in hospital per 1,000,000 people
*weekly_icu_admissions*	Hospital and ICU	Number of COVID-19 patients newly admitted to intensive care units (ICUs) in a given week
*weekly_icu_admissions_per_million*	Hospital and ICU	Number of COVID-19 patients newly admitted to intensive care units (ICUs) in a given week per 1,000,000 people
*weekly_hosp_admissions*	Hospital and ICU	Number of COVID-19 patients newly admitted to hospitals in a given week
*weekly_hosp_admissions_per_million*	Hospital and ICU	Number of COVID-19 patients newly admitted to hospitals in a given week per 1,000,000 people
*stringency_index*	Policy responses	Government Response Stringency Index: composite measure based on 9 response indicators including school closures, workplace closures, and travel bans, rescaled to a value from 0 to 100 (100 = strictest response)
*reproduction_rate*	Reproduction rate	Real-time estimate of the effective reproduction rate (R) of COVID-19
*total_tests*	Test & positivity	Total tests for COVID-19
*new_tests*	Test & positivity	New tests for COVID-19 (only calculated for consecutive days)
*total_tests_per_thousand*	Test & positivity	Total tests for COVID-19 per 1000 people
*new_tests_per_thousand*	Test & positivity	New tests for COVID-19 per 1000 people
*new_tests_smoothed*	Test & positivity	New tests for COVID-19 (7-day smoothed)
*new_tests_smoothed_per_thousand*	Test & positivity	New tests for COVID-19 (7-day smoothed) per 1000 people
*positive_rate*	Test & positivity	The share of COVID-19 tests that are positive, given as a rolling 7-day average (this is the inverse of tests_per_case)
*tests_per_case*	Test & positivity	Tests conducted per new confirmed case of COVID-19, given as a rolling 7-day average (this is the inverse of positive_rate)
*tests_units*	Test & positivity	Units used by the location to report its testing data
*total_vaccinations*	Vaccinations	Total number of COVID-19 vaccination doses administered
*people_vaccinated*	Vaccinations	Total number of people who received at least one vaccine dose
*people_fully_vaccinated*	Vaccinations	Total number of people who received all doses prescribed by the vaccination protocol
*total_boosters*	Vaccinations	Total number of COVID-19 vaccination booster doses administered (doses administered beyond the number prescribed by the vaccination protocol)
*new_vaccinations*	Vaccinations	New COVID-19 vaccination doses administered (only calculated for consecutive days)
*new_vaccionations_smoothed*	Vaccinations	New COVID-19 vaccination doses administered (7-day smoothed)
*total_vaccinations_per_hundred*	Vaccinations	Total number of COVID-19 vaccination doses administered per 100 people in the total population
*people_vaccinated_per_hundred*	Vaccinations	Total number of people who received at least one vaccine dose per 100 people in the total population
*people_fully_vaccinated_per_hundred*	Vaccinations	Total number of people who received all doses prescribed by the vaccination protocol per 100 people in the total population
*total_boosters_per_hundred*	Vaccinations	Total number of COVID-19 vaccination booster doses administered per 100 people in the total population
*new_vaccinations_smoothed_per_million*	Vaccinations	New COVID-19 vaccination doses administered (7-day smoothed) per 1,000,000 people in the total population
*iso_code*	Others	ISO 3166-1 alpha-3–three-letter country/GPE codes
*Continent*	Others	Continent of the geographical location
*Location*	Others	Geographical location
*Date*	Others	Date of observation
*Population*	Others	Population in 2020
*population_density*	Others	Number of people divided by land area, measured in square kilometers, most recent year available
*median_age*	Others	Median age of the population, UN projection for 2020
*aged_65_older*	Others	Share of the population that is 65 years and older, most recent year available
*aged_70_older*	Others	Share of the population that is 70 years and older in 2015
*gdp_per_capita*	Others	Gross domestic product at purchasing power parity (constant 2011 international dollars), most recent year available
*extreme_poverty*	Others	Share of the population living in extreme poverty, most recent year available since 2010
*cardiovasc_death_rate*	Others	Death rate from cardiovascular disease in 2017 (annual number of deaths per 100,000 people)
*diabetes_prevalence*	Others	Diabetes prevalence (% of population aged 20 to 79) in 2017
*female_smokers*	Others	Share of women who smoke, most recent year available
*male_smokers*	Others	Share of men who smoke, most recent year available
*handwashing_facilities*	Others	Share of the population with basic handwashing facilities on premises, most recent year available
*hospital_beds_per_thounsand*	Others	Hospital beds per 1000 people, most recent year available since 2010
*life_expectancy*	Others	Life expectancy at birth in 2019
*human_development_index*	Others	A composite index measuring average achievement in three basic dimensions of human development—a long and healthy life, knowledge, and a decent standard of living

The full dictionary and sources of the dataset are available at https://github.com/owid/COVID-19-data/tree/master/public/data (accessed on 1 December 2021).

**Table A2:** OWID vaccinations by manufacturer dataset dictionary.

Variable	Type	Description
Location	Others	Geographical location
Date	Others	Date of observation
Vaccine	Vaccinations	Manufacturer of the vaccine
total_vaccinations	Vaccinations	Total doses administered. For vaccines that require multiple doses, each individual dose is counted

The full dictionary and sources of the dataset are available at https://github.com/owid/COVID-19-data/tree/master/public/data/vaccinations (accessed on 1 December 2021).
